# Comparing scientific worldviews between allopathic medical degree and East Asian medicine degree students utilizing the thinking about science survey instrument (TSSI)

**DOI:** 10.1186/s12909-021-02956-6

**Published:** 2021-10-29

**Authors:** Saikaew Dudla, Patrick D. Herron, Paul R. Marantz, Felise B. Milan, Corbin Campbell, Belinda J. Anderson

**Affiliations:** 1 Pacific College of Health and Science, 110 William St, New York, NY 10038 USA; 2 Albert Einstein College of Medicine, 1300 Morris Park Ave, Bronx, NY 10461 USA; 3 American University, 4400 Massachusetts Ave, NW, Washington, DC 20016 USA; 4 Pace University, 163 William St, New York, NY 10038 USA

**Keywords:** Complementary and integrative health, Integrative medicine, East Asian medicine, Medical education, Epistemology, Scientific worldviews

## Abstract

**Background:**

Integrative medicine has become a new healthcare model due to the growing evidence base for complementary and integrative therapies. However, some question whether complementary and integrative therapies can truly be integrated with biomedicine due to differences in underlying paradigms and theoretical bases. This study aimed to explore differences in scientific worldviews between students studying East Asian medicine and those completing an allopathic medical degree using the validated Thinking about Science Survey Instrument (TSSI).

**Methods:**

122 medical students from Albert Einstein College of Medicine (Einstein) and 48 East Asian medicine students from the Pacific College of Health and Science (Pacific College) participated in this study. Participants completed the TSSI, a 60-item Likert-scale instrument that quantitatively measures the sociocultural resistance to, and support for science. Item and category means were compared between each group using an independent sample t-test.

**Results:**

Distinct differences were seen between the two groups of students with regard to age, gender distribution and prior education. Einstein students were generally supportive of science and Pacific College students were generally supportive of/positively neutral to science. Einstein students more strongly affirmed the relationship of science in relation to the categories of Epistemology, Public Health, Emotion and Aesthetics, the Economy, and Public Policy. Pacific College students more strongly affirmed the relationship between science and the category Race and Gender. There were no differences in the categories of Environment and Resource, Science for All, and Religion and Morality.

**Conclusion:**

This study suggests that there are differences underlying the scientific worldviews of Einstein and Pacific College students, particularly with regard to Epistemology and Public Health. Such differences may be related to the different theoretical knowledge bases and ways of viewing health within the two disciplines. Despite demographic and educational differences between the two groups their overall scientific worldviews were similar with neither group expressing disparate views. This suggests that both groups may be receptive to the value of other paradigms. Providing courses that focus on different therapeutic approaches and paradigms during medical training may foster interprofessional understanding and collaborative practice between health professionals of different medical disciplines.

## Introduction

Over the past several decades there has been a growing interest in complementary and integrative health (CIH), an approach that encompasses many practices, interventions and products outside the origin of conventional Western medicine (biomedicine) [1]. The National Institutes of Health (NIH), National Center for Complementary and Integrative Health (NCCIH) categorizes CIH approaches into “mind-body practices” (e.g. acupuncture, yoga, chiropractic, etc.) and “natural products” (e.g. herbs, vitamins, supplements etc.), and refers to them collectively as Complementary Health Approaches [1]. The healthcare model in which CIH and biomedicine are practiced together is called integrative medicine.

There have been many interprofessional education initiatives in biomedical and CIH institutions. In 2000, the NCCIH, through the R25 grant mechanism (the Complementary and Alternative Medicine Educational Project), funded 15 biomedical institutions to incorporate CIH into their degree programs [2]. A second round of NIH R25 educational grants (the Complementary and Alternative Medicine Practitioner Research Education Project Grant Partnership) enabled nine CIH institutions to implement research and evidence-based medicine into their degree programs, in partnership with a research-intensive institution [3].

One such interprofessional education initiative is the interprofessional education student exchange program partnership between Albert Einstein College of Medicine (Einstein) and Pacific College of Health and Science (Pacific College; formerly Pacific College of Oriental Medicine) [4]. Participating students engage in interprofessional learning activities inclusive of lectures, clinical observerships, and dissection laboratory classes.

Despite the public demand [5, 6] and rise in popularity of integrative medicine, some question whether the vastly different paradigms of CIH and biomedicine can truly be integrated [7]. CIH is derived from prescientific theories and philosophies, while biomedicine is based on bioscience [8]. Since biomedicine is the dominant medicine in the United States, some argue that CIH cannot maintain its integrity if it becomes coopted and “reconstructed on a scientific foundation.” [8, 9] Previous studies have indicated that although CIH students found science and the scientific method valuable, they were wary of its relevance to clinical practice [10, 11]. In particular, they viewed the scientific method’s reductionist approach, such as the randomized controlled trial (RCT), as incapable of assessing a holistic medical system such as East Asian medicine [9].

Issues have also been raised about the value of CIH education in medical degree programs. One study reported that CIH education was thought to be valuable due to providing a more holistic perspective to patient care [12]. However, questions arise as to which CIH therapies should be incorporated into an already dense medical curriculum, especially considering the inconsistencies in scientific evidence supporting the efficacy and effectiveness of the various CIH therapies [2]. Another concern is to extent to which medical students would be expected to incorporate CIH education into their clinical practice [2].

An aspect likely related to the challenges associated with integrating biomedicine with the complementary and integrative therapies, especially those based on fundamentally different paradigms, are the worldviews held by the practitioners of the different medical systems. Using the validated Thinking about Science Survey Instrument [13] we surveyed students participating in the interprofessional exchange program between Einstein and Pacific College, to inquire about their scientific worldviews in relation to various social and cultural factors.

Understanding students’ perspectives is especially important as they represent the future of our healthcare system. Through this study, we aim to provide a quantitative narrative on how these two groups may value science differently in the context of important socio-cultural issues, and how these differences can be overcome as we face increasing integration of different healthcare disciplines.

## Methods and materials

### Sampling

A convenience sampling technique was used. The annual Einstein-Pacific College interprofessional exchange program allows students from the respective institutions to participate in a non-graded elective. These include Einstein students in their first year of the four-year medical degree program, and Pacific College students in their final year of the three year and eight-month Master’s degree program. This cohort of 170 Einstein and 109 Pacific College students were invited to participate in the study.

### IRB and survey implementation

Use and implementation of these surveys was approved by the Einstein and Pacific College institutional review boards, and informed consent was obtained from all participants. All methods were carried out in accordance with relevant guidelines and regulations. Surveys were undertaken online through Einstein’s Research Electronic Data Capture (REDCap) system. This system housed the surveys, sent invitation emails, and stored outcome data. Emails with a link to the survey came from PH (the Co-Director for Einstein’s Introduction to Clinical Medicine (ICM) Program when the study was undertaken) to the Einstein students, and from BA (the Academic Dean at Pacific College when the study was undertaken) to the Pacific College students. An initial email followed by two reminder emails were sent one week apart. Students were informed that survey participation was voluntary with no penalty for non-participation. The interprofessional program is a non-mandatory, ungraded part of the degree program at both institutions, and there is no penalty for non-participation. Students were offered a $10 Amazon gift card for completing the survey as well as being entered into a lottery to win an Apple iPad.

### Survey and TSSI

This study utilized the Thinking about Science Survey Instrument (TSSI) with no modifications being made to the original instrument. The TSSI was preceded by demographic questions and questions that allowed participant tracking. The TSSI is a validated tool intended to quantitatively measure how subjects value science with regard to other socio-cultural aspects of society [13]. It was originally developed to measure preservice elementary teachers’ attitudes towards science, with the concern that the public at large, including elementary teachers, resist the paradigm of science, and view science incongruently with their personal beliefs about other areas of life [13]. In addition to the original population, the TSSI has also been used to study attitudes towards science held by the general U.S. public [14] and undergraduate university students [15, 16].

The TSSI consists of 60 items containing a statement expressing a particular view about science in relation to other areas of culture and society. Question responses use a standard 5-point Likert agree/disagree scale. Each of the sixty items measures the respondent’s view against a “common image of science,“ [13] and falls into one of nine categories representing different aspects of society. The creator of the TSSI stated that “these categories are not intended to represent an authoritative scientific worldview, but a scientific worldview version commonly found in both the popular media and the literature of science and science education.” [13]

Twenty-seven items required reverse scoring to create consistency when included as part of a category because they contain negatively worded questions for the resistance to science. Reversed questions have been indicated with ‘R’ in the results table, with the items presented with their original wording. While reverse scoring was used for analyses performed within categories, individual item means reflect the scores assigned to the item as worded (i.e., non-reversed).

### Data analysis

The independent sample t-test was performed to compare the Einstein and Pacific College students’ mean scores for each survey item and the mean scores for each of the nine survey categories [17]. The score at the category level was computed by taking the average of the subject’s scores across all items in that category. A two-tailed *p* < 0.05 was considered statistically significant.

## Results

### Demographics

Demographic data is shown in Table 1. The response rate was 72% (*n* = 122) for Einstein students and 44% (*n* = 48) for Pacific College students. There were distinct differences between the two groups with respect to gender, age, and prior education. The Einstein students had a relatively even distribution of male and female students, whereas the Pacific College students were predominantly female. The latter group was also generally older than the former. Most students at both institutions held a bachelor’s as their highest degree, although Pacific College contained more diversity among the remaining degree possibilities.

**Table 1:** Demographic data

Questions		*Medical students* *N (%)*	*East Asian medicine degree students* *N(%)*
**Response Rate**	Surveys Sent	170 (100)	109 (100)
	Total Responded	122 (72)	48 (44)
**Gender**	Answered	119 (100)	47 (100)
	Male	63 (52.9)	7 (14.9)
	Female	54 (45.4)	39 (83)
	Transgender	0 (0)	1 (2.1)
	Other	2 (1.7)	0 (0)
**Age**	Answered	119 (100)	47 (100)
	Prefer not to answer	3 (2.5)	3 (6.4)
	20–25	106 (89.2)	6 (12.8)
	26–30	8 (6.6)	14 (29.8)
	31–50	2 (1.7)	19 (40.4)
	51+	0 (0)	5 (10.6)
**Highest Level of Education**	Answered	120 (100)	46 (100)
	High School	0 (0)	1 (2.2)
	Associates	0 (0)	5 (10.9)
	Bachelors	115 (95.8)	30 (65.2)
	Masters	5 (4.2)	7 (15.2)
	Doctoral	0 (0)	3 (6.5)
**Previous Area of Study/Studies**	Answered	156 (100)	60 (100)
	Natural Sciences	87 (55.7)	20 (33.3)
	Social Sciences	37 (23.7)	13 (21.7)
	Engineering	14 (9)	0 (0)
	Humanities	14 (9)	6 (10)
	Other	4 (2.6)	21 (35)

### TSSI survey

Table 2 shows the nine TSSI categories and their corresponding item means for the two student groups, ordered by smallest *p*-value. The criteria for alignment with the model (supportive of science) is a score between 3.51 and 5.00, whereas neutral to the model is between 2.51 and 3.50, and against the model (resistance to science) is between 1.00 and 2.50. Of the 60 items within the nine broad TSSI categories, 22 showed statistically significant differences between the Einstein and Pacific College students.

**Table 2:** TSSI category and item mean comparison between Einstein and Pacific College students

TSSI Category and Corresponding Items	Mean (SD)		P-value
	*Einstein students* *(n = 122)*	*Pacific College students* *(n = 48)*	
**Epistemology: Science is a superior, exemplary form of knowledge that produces highly reliable and objective knowledge about the real world.**	**3.07**^**b**^ **(0.64)**	**2.61**^**b**^ **(0.71)**	<.0001*
33. The methods of science are the most reliable source of true, factual knowledge.	3.66^a^ (1.00)	2.81^b^ (0.91)	<.0001*
34. Science is the best source of reliable knowledge.	3.75^a^ (1.04)	2.90^b^ (0.95)	<.0001*
17. Scientific knowledge is the most objective form of knowledge.	3.81^a^ (1.00)	3.06^b^ (1.14)	<.0001*
60. Scientific knowledge is the truest form of knowledge.	3.32^b^ (1.10)	2.67^b^ (1.23)	0.001*
46. The methods of science are objective.	3.48^b^ (0.88)	3.13^b^ (0.94)	0.02*
2. No source of knowledge provides absolute truth - not even science.^R^	3.50^b^ (1.22)	3.83^a^ (1.14)	0.1
29. We can be certain that scientific knowledge is reliable.	2.81^b^ (1.05)	2.63^b^ (1.16)	0.32
44. No form of knowledge - including science - can ever be completely objective.^R^	3.85^a^ (0.97)	3.96^a^ (0.92)	0.52
27. No form of knowledge can be completely certain - not even scientific knowledge.^R^	3.88^a^ (1.09)	3.88^a^ (1.12)	0.96
**Public Health: The conquering of disease and physical affliction and the great advances in public health are made possible by science and will not continue without science.**	**3.93**^**a**^ **(0.58)**	**3.49**^**b**^ **(0.55)**	<.0001*
8. Scientific knowledge is the single most important factor in the improvement of medicine and public health.	3.26^b^ (1.26)	2.60^b^ (1.20)	0.002*
9. Common sense contributes more to good health than does scientific knowledge.^R^	2.64^b^ (1.00)	3.10^b^ (0.93)	0.01*
58. Scientific knowledge contributes little to good health.^R^	1.52^c^ (1.01)	1.92^c^ (1.03)	0.02*
48. Scientific research makes important contributions to medicine and the improvement of public health.	4.62^a^ (0.67)	4.38^a^ (0.73)	0.04*
**Emotion and Aesthetics: Scientists are often passionate about their work but the work of science best proceeds on the basis of objective reason and empiricism. There is a beauty to science. Indeed, “elegance” is often required of scientific ideas.**	**3.80**^**a**^ **(0.50)**	**3.49**^**b**^ **(0.49)**	0.0004*
12. Scientific explanations tend to spoil the beauty of nature.^R^	1.50^c^ (0.82)	2.13^c^ (1.08)	0.001*
21. It is equally important for a person to have scientific knowledge and an appreciation for the arts.^R^	3.74^a^ (1.10)	4.27^a^ (0.94)	0.004*
36. Science can contribute to our appreciation and experience of beauty.	4.50^a^ (0.76)	4.15^a^ (0.62)	0.005*
1. Human emotion plays no part in the creation of scientific knowledge.^R^	2.07^c^ (1.07)	1.77^c^ (1.02)	0.1
**Economy: Modern industrial, commercial, and information-based economies depend on the scientific developments for increasing production, wealth and general public welfare.**	**4.16**^**a**^ **(0.42)**	**3.92**^**a**^ **(0.41)**	0.0008*
42. Science is our best source of useful knowledge.	3.69^a^ (1.01)	2.79^b^ (1.11)	<.0001*
22. The development of our natural resources, such as coal, gas, oil, solar energy, requires much more than scientific knowledge.^R^	3.62^a^ (0.99)	3.96^a^ (0.85)	0.04*
49. Developing new scientific knowledge is very important for keeping our country economically competitive in today’s world.	4.41^a^ (0.66)	4.15^a^ (0.87)	0.06
25. There are many good things we can do today because of scientific knowledge.	4.70^a^ (0.62)	4.50^a^ (0.74)	0.08
51. Scientific knowledge is useful.	4.71^a^ (0.60)	4.56^a^ (0.62)	0.14
14. The strength of our national economy does not depend on scientific knowledge.^R^	1.83^c^ (0.92)	2.04^c^ (0.85)	0.17
31. The development of our natural resources, such as coal, gas, oil, solar energy, is dependent upon having adequate scientific knowledge.	4.33^a^ (0.62)	4.15^a^ (0.82)	0.17
20. Scientific knowledge is useful in keeping our national economy competitive in today’s world.	4.17^a^ (0.75)	4.06^a^ (0.86)	0.41
41. Scientific knowledge is useful for only a few people.^R^	1.47^c^ (0.63)	1.54^c^ (0.80)	0.56
16. Science helps develop our natural resources such as coal, gas, oil, and solar energy.	4.49^a^ (0.71)	4.50^a^ (0.77)	0.95
**Public Policy: Science acts in the public interest. Science should thus be supported by public funds, however, the science community is more than capable of policing scientific activity.**	**3.29**^**b**^ **(0.36)**	**3.10**^**b**^ **(0.38)**	0.0027*
50. Scientific knowledge influences government decision making too much.^R^	2.06^c^ (0.82)	2.83^b^ (0.97)	<.0001*
6. Scientific research is generally very important.	4.61^a^ (0.64)	4.25^a^ (0.84)	0.01*
57. The government should not be in the business of using tax dollars to fund scientific research.^R^	1.59^c^ (0.86)	1.96^c^ (1.05)	0.02*
5. Scientific research is rarely dangerous to the public.	2.72^b^ (1.04)	2.33^c^ (1.12)	0.04*
10. Scientific research should be adequately funded by government.	4.58^a^ (0.68)	4.38^a^ (0.67)	0.07
45. Scientific research is economically and politically determined.^R^	3.98^a^ (0.74)	3.75^a^ (0.89)	0.08
28. Scientific research should be carefully regulated by law.^R^	3.57^a^ (1.06)	3.69^a^ (1.06)	0.52
26. Scientists should not be allowed to research anything they wish.^R^	2.79^b^ (1.13)	2.71^b^ (0.92)	0.64
18. Scientific research is often potentially dangerous to the public.^R^	2.75^b^ (1.04)	2.75^b^ (1.02)	0.98
19. There is little need for the legal regulation of scientific research.	1.71^c^ (0.83)	1.71^c^ (0.87)	0.99
**Race and Gender: Science is an “equal opportunity employer.” Race, gender and other personal factors are irrelevant in science.**	**2.87**^**b**^ **(0.95)**	**3.22**^**b**^ **(1.09)**	0.0395*
23. The scientific community is mostly dominated by men and is often unfriendly to women.^R^	3.35^b^ (0.98)	2.92^b^ (1.13)	0.01*
53. The scientific community is mostly dominated by white men and is often unfriendly to minority people.^R^	3.31^b^ (1.07)	2.88^b^ (1.21)	0.02*
30. African Americans and other minority people are just as welcome in the scientific community as are white people.	2.92^b^ (1.31)	3.23^b^ (1.49)	0.18
4. Women are welcome in science just as much as men are.	3.22^b^ (1.38)	3.44^b^ (1.43)	0.36
**Environment and Resource: Science is necessary for the discovery, development, and conservation and protection of natural resources and the environment in general.**	**3.84**^**a**^ **(0.58)**	**3.66**^**a**^ **(0.70)**	**0.0981**
43. Science can help us preserve our natural environment and natural resources.	4.40^a^ (0.65)	4.10^a^ (0.99)	0.06
59. Without science we will not be able to preserve our natural environment and natural resources.	4.02^a^ (0.91)	3.65^a^ (1.26)	0.07
38. Our natural environment would actually be helped by the absence of scientific knowledge.^R^	1.78^c^ (0.96)	1.94^c^ (0.91)	0.33
3. Scientific knowledge has often contributed to the destruction of our environment and natural resources.^R^	3.30^b^ (1.20)	3.17^b^ (1.39)	0.55
**Science for All: The importance of science is such that it should be taught at all levels of schooling. Every citizen should have attained at least a minimal level of science literacy.**	**4.07**^**a**^ **(0.56)**	**4.17**^**a**^ **(0.52)**	**0.2918**
13. Students should not be forced to take science courses at the university.^R^	2.20^c^ (1.18)	1.65^c^ (0.84)	0.001*
55. Even at the university level all students should study at least some science.	3.84^a^ (1.02)	4.31^a^ (0.85)	0.01*
54. Most people really do not need to know very much science.^R^	2.34^c^ (1.04)	2.04^c^ (0.92)	0.08
37. Only a very few people really understand science.^R^	2.43^c^ (0.99)	2.73^b^ (1.14)	0.1
15. Science should not be made an important subject for the elementary school grades.^R^	1.40^c^ (0.92)	1.56^c^ (1.03)	0.32
52. All students should study science during the secondary school grade levels.	4.45^a^ (0.74)	4.35^a^ (0.84)	0.46
56. Science should be taught at all school grade levels.	4.22^a^ (0.97)	4.33^a^ (0.81)	0.49
24. Understanding science is a good thing for everyone.	4.42^a^ (0.75)	4.33^a^ (0.91)	0.52
**Religion and Morality: People make moral choices about the use of scientific findings but science itself is morally neutral. Science is also neutral with regard to religion. The importance of science, however, is such that science must be protected from the intrusive activities of some religions.**	**2.68**^**b**^ **(0.72)**	**2.60**^**b**^ **(0.54)**	**0.5211**
11. Science is a more important source of knowledge than religion.	3.61^a^ (1.26)	3.19^b^ (1.27)	0.053
32. Religious knowledge contributes more to the well being of a person’s life than does science.^R^	2.43^c^ (0.98)	2.67^b^ (0.95)	0.15
47. Scientific knowledge tends to erode spiritual values.	2.33^c^ (0.93)	2.50^c^ (1.07)	0.3
7. A person can be both religious and scientific.^R^	4.34^a^ (0.87)	4.48^a^ (0.74)	0.33
39. Religion and science are almost always at odds with each other.	2.25^c^ (1.10)	2.35^c^ (1.04)	0.59
35. Scientific research is morally neutral.	2.56^b^ (1.08)	2.58^b^ (1.11)	0.91
40. Religion tends to impede scientific progress.	2.77^b^ (1.25)	2.75^b^ (1.08)	0.92

^R^ Negatively worded statement—means were not reversed

^a^ Align with the model

^b^ Neutral to the model

^c^ Reject the model

^*^ p < 0.05

**Fig. 1:**
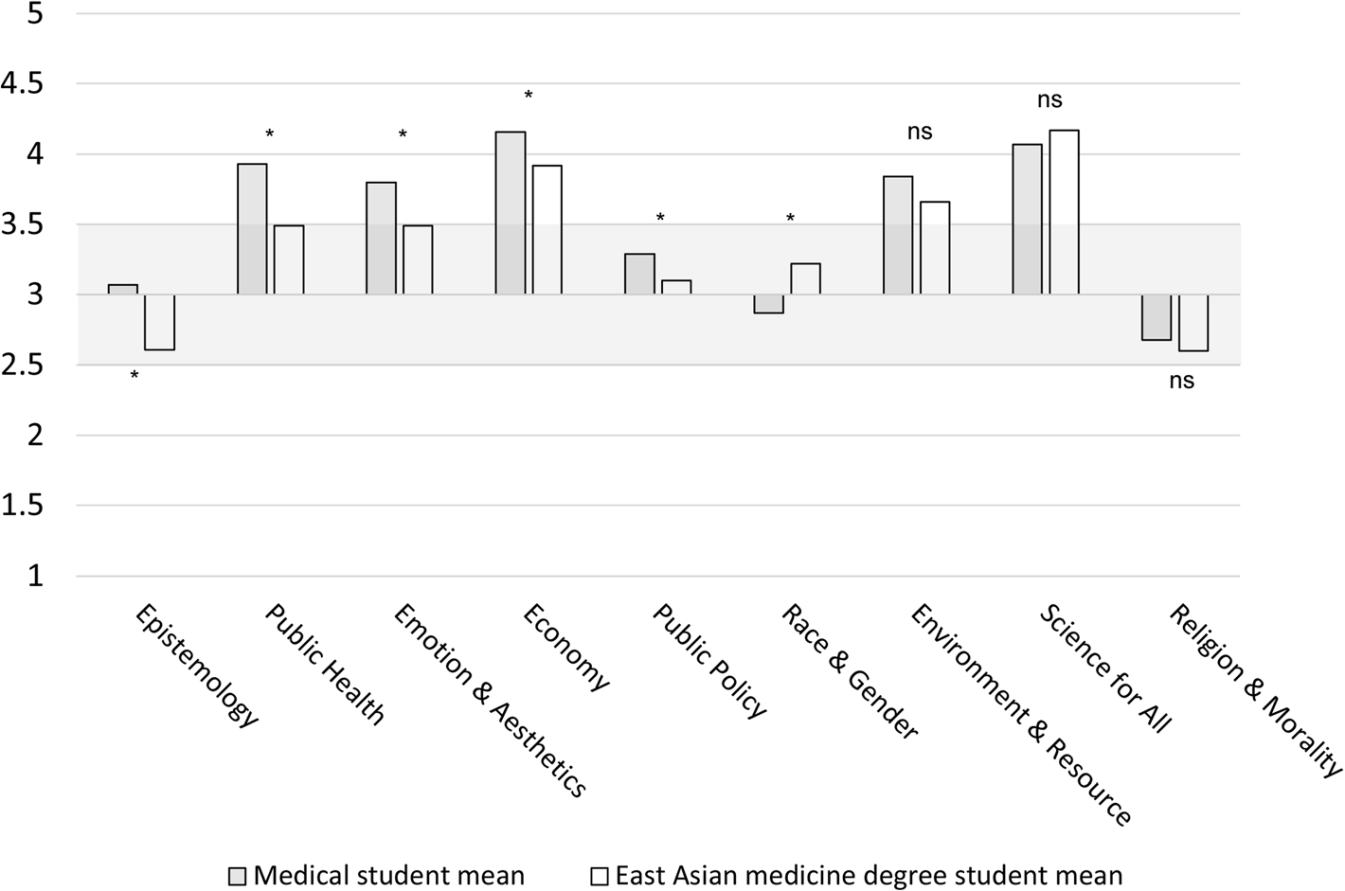
Category means of allopathic medical degree students and East Asian medicine degree students. Legend: Category means of medical degree students (n = 122), represented as grey columns and East Asian medicine degree students (n = 48), represented as white columns and ordered (from left to right) by smallest p-value. The horizontal pale grey shaded area represents the neutral score range. The area above the neutral range represents scores that are consistent with the model (greater sociocultural support for science), and below as inconsistent with the model

Figure 1 visually depicts the data in Table 2. The x-axis contains the 9 categories, and the y-axis contains scores ranging from 1 to 5. Each bar represents the mean score per category, with grey bars representing Einstein means and white bars representing Pacific College student means. The horizontal pale grey shaded area represents the neutral score range. The area above the neutral range represents scores that are consistent with the model (greater sociocultural support for science), and below as inconsistent with the model. From this figure, it is evident that neither group is ‘anti-science’ in any of the TSSI categories, with the majority of scores being above neutral or on the positive end of the neutral range. The nine categories and their items will be discussed in more detail below, by order of the category with the greatest statistically significant difference between the student groups.

#### Epistemology category

(“Science is a superior, exemplary form of knowledge that produces highly reliable and objective knowledge about the real world.” [13])

In the Epistemology category Einstein students were neutral (M = 3.07, SD = 0.64), and Pacific College students were barely neutral (M = 2.61, SD = 0.71), scoring significantly lower than the Einstein students (*p*-value <.0001). In five of nine items in the Epistemology category the two groups showed significant differences (Table 2), and Einstein students scored higher on all five of these items.

#### Public health category

(“The conquering of disease and physical affliction and the great advances in public health are made possible by science and will not continue without science.” [13])

In the Public Health category Einstein students showed a significantly greater level of agreement (M = 3.93, SD = 0.58), compared to Pacific College students who bordered the upper neutral zone (M = 3.49, SD = 0.55; *p*-value <.0001). The students exhibited significant differences in all four Public Health items (Table 2).

#### Emotion and aesthetics category

(“Scientists are often passionate about their work but the work of science best proceeds on the basis of objective reason and empiricism. There is a beauty to science. Indeed, “elegance” is often required of scientific ideas.” [13])

In the Emotion and Aesthetics category Einstein students scored above the neutral zone with a mean of 3.80 (SD = 0.50), whereas Pacific College students were neutral with a mean of 3.49 (SD = 0.49), and the difference was significantly different (*p*-value = 0.0004). Among the four items in this category, three were significantly different (Table 2).

#### Economy category

(“Modern industrial, commercial, and information-based economies depend on the scientific developments for increasing production, wealth and general public welfare.” [13])

Both groups aligned with the model (Einstein M = 4.16, SD = 0.42, Pacific College M = 3.92, SD = 0.41), although the difference was significant (*p*-value = 0.0008). This is the highest ranked category for the Einstein students and the second highest ranked for the Pacific College students. Two out of the 10 items in this category showed significant differences between the two groups (Table 2).

#### Public policy category

(“Science acts in the public interest. Science should thus be supported by public funds, however, the science community is more than capable of policing scientific activity.” [13])

In the Public Policy category both groups were neutral (Einstein M = 3.29, SD = 0.36, Pacific College M = 3.10, SD = 0.38), although Einstein students scored significantly higher (*p*-value = 0.0027). Among the 10 items in this category, four were significantly different between the two student groups (Table 2).

#### Race and gender category

(“Science is an “equal opportunity employer.” Race, gender and other personal factors are irrelevant in science.” [13])

Although both groups scored in the neutral range in this category, Einstein students fell on the negative side of neutral (M = 2.87, SD = 0.95), and Pacific College students fell on the positive side of neutral (M = 3.22, SD = 1.09). These differences were statistically significant (*p*-value = 0.0395). Two out of the four items were significantly different (Table 2).

#### Environment and resource category

(“Science is necessary for the discovery, development, and conservation and protection of natural resources and the environment in general.” [13])

In the Environment and Resource category both groups scored above the neutral range (Einstein M = 3.84, SD = 0.58, Pacific College M = 3.66, SD = 0.70), and there was no significant difference between the two groups for the category mean (*p*-value = 0.0981) or the four item means.

#### Science for all category

(“Science for All: The importance of science is such that it should be taught at all levels of schooling. Every citizen should have attained at least a minimal level of science literacy.” [13])

In the Science for All category both groups scored above the neutral range (Einstein M = 4.07, SD = 0.56, Pacific College M = 4.17, SD = 0.52). The difference between the mean scores for the two groups was not significantly different (p-value = 0.2918).

#### Religion and morality category

(“People make moral choices about the use of scientific findings but science itself is morally neutral. Science is also neutral with regard to religion. The importance of science, however, is such that science must be protected from the intrusive activities of some religions.” [13])

In the Religion and Morality category both groups scored in the negative range of neutral, with a mean of 2.68 (SD = 0.72) for Einstein and 2.60 (SD = 0.54) for Pacific College. The mean difference for the category, and for all of the seven items was not significantly different between the two student groups (*p*-value = 0.5211).

## Discussion

This study evaluated and compared the scientific worldview differences between medical degree and East Asian medicine students. Our results indicate that, of the nine categories, Einstein students were overall supportive of science, whereas Pacific College students were supportive of/positively neutral to science. Einstein students more strongly affirmed the relationship of science in relation to the categories of Epistemology, Public Health, Emotion and Aesthetics, the Economy, and Public Policy. Pacific College students more strongly affirmed the relationship between science and the category of Race and Gender. There were no differences in the categories of Environment and Resource, Science for All, and Religion and Morality.

Several important trends were seen in the demographic data. The Einstein students were almost evenly distributed between male and female whereas the Pacific College students were predominantly female. These numbers similarly reflect the gender distribution in their respective institutions [18, 19]. The Einstein students were more than twice as likely to have studied the natural sciences before matriculating to medical school, as compared to the Pacific College students. This difference is most likely due to the basic science prerequisite coursework needed to apply to a United States medical school, a requirement not shared in US East Asian medicine schools. This would likely have imparted greater scientific knowledge for Einstein students and may explain why they are more supportive of science overall. The Pacific College students were generally older than the medical students, possibly because the students surveyed included 1st year medical students and senior year East Asian medicine degree students. Another possible explanation for this may be that many students of East Asian medicine in the US pursue this field as a second career and/or have completed graduate school training, therefore matriculating at Pacific College at a later age than most medical students who pursue a medical degree immediately after completing their undergraduate degree. Overall, the demographics data indicate that these are two contrasting group of learners with distinct characteristics in age, sex, level of prior education, previous area studied, and who enter their professional education at different stages of development. Despite these differences, their general scientific worldviews are not completely disparate of each other and show many similarities.

Both groups of students endorsed the crucial role science plays in facilitating many advancements and improvements to society, as reflected in their affirmations that “scientific knowledge is useful” (item 51) and that “there are many good things we can do today because of scientific knowledge” (item 25). As upcoming healthcare professionals, both groups appreciated the value science has in regard to public health, affirming that “scientific research makes important contributions to medicine and the improvement of public health” (item 48). For the Pacific College students, this suggests that subscribing to a paradigm that is different than biomedicine does not equate to anti-science beliefs, and counters the stereotype that has sometimes been used to describe CIH practitioners as “quacks.” [20]

There appears to be fundamentally different epistemological perspectives between the two groups of students, as suggested by the differences in the Epistemology category. These distinctions likely arise from the different theoretical knowledge base of the two disciplines. East Asian medicine builds from three conceptual principles - yin and yang, the five elements and qi [21, 22]. These abstract and immaterial principles contrast heavily with biomedicine, which builds on the natural sciences, such as biology and biochemistry.

These contrasting worldviews have established vastly different methodologies in obtaining knowledge. The methods of biomedicine “seeks to discover the objective truth about the natural world via framing hypotheses and subjecting them to rigorous experimental tests under controlled conditions.” [8] The randomized controlled trial (RCT), which has undoubtedly made important contributions to modern medicine, is the representative method of assessing therapeutic interventions, because of its design to reduce bias. Einstein students support this model, affirming that “Scientific knowledge is the most objective form of knowledge” and therefore “Science is the best source of reliable knowledge” (Epistemology, items 17 and 34). This has important implications because studies have shown that medical students and physicians rely on the results of RCTs and mechanistic studies to form an opinion about CIH [23, 24].

Historically East Asian medicine had relied upon information on the lowest tiers of the evidence hierarchy [25] such as classical texts and expert opinion and experience to inform their practice. To meet modern scientific scrutiny, many RCTs have been conducted to study East Asian medicine interventions, but they have often provided inconclusive and disappointing results due to methodological designs that fail to study the interventions in their original context [26]. Acupuncture RCTs have also struggled to produce an inert placebo-control, which brings into question whether they achieved the goal of minimalizing therapeutic effects [27]. This may explain why the largest significant mean difference in Epistemology was on item 33, “The methods of science are the most reliable source of true, factual knowledge.” Pacific College students were neutral in response to this statement, whereas Einstein students were supportive. Awareness of the inability of some scientific methodological approaches to encompass the complexity of East Asian medical interventions, and make evidence-based clinical inferences, may have influenced their perspectives in relation to this statement. Alternative methodologies such as mixed-methods, whole-system approaches, and pragmatic clinical trials have been recommended to more accurately assess CIH interventions [28, 29].

The fact that the Einstein students scored in the neutral range in the Epistemology category suggests that although they believe science is the most ideal method of knowledge creation (items 17, 34, 33), it has limitations (items 2, 44, 27). This suggests that Einstein students may have awareness of and/or may even potentially be open to other worldviews, a notion presented in previous studies. In one medical school where students undertook a mandatory CIH course, the students appeared “willing to challenge their pre-existing beliefs about medicine.” [12] In another study that explored physicians’ views, those who had positive views of CIH often had a history of exposure to CIH, which likely prompted a shift in their worldview, with one physician stating that CIH “described human nature in a way that I could recognize.” [30]

The Pacific College students’ neutrality in the Public Health category may be related to their perception that the holistic aspects of East Asian medicine are not fully compatible with the more reductionist approach of biomedicine. The emotional, spiritual and cultural determinants of health are central to East Asian medicine. In many ways this is conceptually similar to the biopsychosocial model of care [31].

Although the Einstein students affirmed the Public Health category overall, they expressed neutrality to the statement “Scientific knowledge is the single most important factor in the improvement of medicine and public health” (Public Health, item 8), and this item had the highest degree of variance in this category. This variability may reflect the gradual societal acceptance of the biopsychosocial model and the social determinants of health, and the fact that Einstein endorses this model and introduces it early in their medical curriculum. Previous studies have shown that medical students and physicians were aware of CIH as a distinct model of care that embraces holistic, self-care and behavioral change [12, 32]. The Einstein students were also more opposed to the statement that “Common sense contributes more to good health than does scientific knowledge” (Public Health, item 9). This could suggest that while common sense can contribute to good health, it may cause physicians to miss important diagnoses if taken in lieu of more sophisticated procedures such as imaging and blood tests.

The remaining categories contain other important group- and item-level distinctions concerning the contextual valuation of science. Taken as a whole, our data suggests that Einstein students are generally more favorable of science with regard to broader areas of society indirectly related to medicine. One exception is in Race and Gender category, where the Pacific College students perceived the scientific community to be friendlier towards women and minorities than the Einstein students. This view may be a reflection of their comparably limited exposure to the field of academic medicine.

An unexpected finding in the Science for All category was that Pacific College students more strongly affirmed that university-level students should study at least some science. Previous studies on Pacific College students have shown they highly valued scientific research to elevate the profession and improve patient care [33]. This utilization of scientific research could explain their advocacy for university-level scientific education.

There are several limitations to this study. The specific institutions from which the participants were sampled may not represent all allopathic medical and East Asian medicine institutions in the US. Einstein’s medical degree program emphasizes the humanistic and compassionate components of patient care, a holistic philosophy that most likely attracted students with similar values. Pacific College’s East Asian medicine degree program has a strong research and evidence-based medicine component woven throughout its curriculum, which may not exist in other East Asian medicine institutions. Einstein and Pacific College are institutions based in New York City, and students who opted to live in this geographical location may be different compared to students in the rest of the US. We did not collect racial and ethnic data, which may have provided insight into some of the outcomes. Students at the two institutions were at different stages of their respective degree programs, which may have contributed to some of the differences in their scientific perspectives.

Another limitation was the low response rate in Pacific College students (44%), which may have caused response bias. In general, the Pacific College students are older and therefore may have lower rates of computer and internet literacy/usage, possibly leading to lower response rates [34]. It is also known that response rates tend to be higher when the participants find the survey topic interesting [34, 35]. Since the TSSI deals with scientific worldviews, it is possible that Pacific College non-respondents were not interested in the topic. This may have produced data indicating a more positive scientific worldview perceived by the Pacific College students. Overall, Einstein respondent demographics did not greatly differ from the entire class of 2020, so we can’t point to evidence of response bias. However, there is not enough information from Pacific College non-responders to infer a lack of non-response bias.

We did not provide the opportunity for free responses, which would have justified and clarified the answers chosen by the students. Gathering more qualitative data on these areas could perhaps be undertaken in a future study. Lastly, this study was undertaken before the coronavirus pandemic, which likely would have impacted the results of the study.

## Conclusions

This study suggests that there are differences underlying the scientific worldviews of Einstein and Pacific College students, with the largest differences in the Epistemology category. This finding may be a reflection of the paradigms their respective medicines hold and can present a barrier to integration. Considering that over 30% of the U.S. adult population uses any CIH therapy [6], our study underscores the importance of including curriculum specific to approaches to healthcare that are based on different paradigms. Despite the difference in worldviews, neither group of students hold disparate views for or against science. Our study therefore suggests that upcoming medical degree and East Asian medicine degree students may be receptive to learning perspectives besides their own. Worldview consciousness is an important clinical skill to facilitate communication and receptiveness, and to reduce implicit bias [36]. To foster this skill, courses on different beliefs, cultures and worldviews should be taught early in medical degree and East Asian medicine degree curriculums to encourage collaboration and communication between biomedical and CIH students [4, 36].

## Data Availability

The datasets used and/or analyzed during the current study are available from the corresponding author on reasonable request.

## Abbreviations

BA: Belinda Anderson
CC: Corbin Campbell
CIH: Complementary and Integrative Health
Einstein: Albert Einstein College of Medicine
FM: Felise Milan
NIH: National Institutes of Health
NCCIH: National Center for Complementary and Integrative Health
Pacific College: Pacific College of Health and Science
PH: Patrick Herron
PM: Paul Marantz
RCT: Randomized Controlled Trial
REDCap: Research Electronic Data Capture
SD: Saikaew Dudla
TSSI: Thinking about Science Survey Instrument
